# In Situ Formation of Decavanadate-Intercalated Layered Double Hydroxide Films on AA2024 and their Anti-Corrosive Properties when Combined with Hybrid Sol Gel Films

**DOI:** 10.3390/ma10040426

**Published:** 2017-04-18

**Authors:** Junsheng Wu, Dongdong Peng, Yuntao He, Xiaoqiong Du, Zhan Zhang, Bowei Zhang, Xiaogang Li, Yizhong Huang

**Affiliations:** 1 Institute of Advanced Materials and Technology, University of Science and Technology Beijing, Beijing 100083, China; wujs@ustb.edu.cn (J.W.); dxheyq@163.com (X.D.); yxzz0417@163.com (Z.Z.); lixiaogang99@263.net (X.L.); 2 School of Materials Science and Engineering, Nanyang Technological University, Singapore 639798, Singapore; BZHANG011@e.ntu.edu.sg; 3 National Science &Technology Infrastructure Center, Beijing 100862, China; wujsyy@126.com

**Keywords:** layered double hydroxides (LDHs), aluminum alloy, corrosion protection, scanning electrochemical microscopy (SECM)

## Abstract

A layered double hydroxide (LDH) film was formed in situ on aluminum alloy 2024 through a urea hydrolysis method, and a decavanadate-intercalated LDH (LDH-V) film fabricated through the dip coating method. The microstructural and morphological characteristics were investigated by scanning electron microscopy (SEM). The corrosion-resistant performance was analyzed by electrochemical impedance spectroscopy (EIS), scanning electrochemical microscopy (SECM), and a salt-spray test (SST).The SEM results showed that a complete and defect-free surface was formed on the LDH-VS film. The anticorrosion results revealed that the LDH-VS film had better corrosion-resistant properties than the LDH-S film, especially long-term corrosion resistance. The mechanism of corrosion protection was proposed to consist of the self-healing effect of the decavanadate intercalation and the shielding effect of the sol-gel film.

## 1. Introduction

The traditional method of protecting aluminum alloy 2024 (AA2024), which has been universally employed in various industries, consisted of chromate conversion coatings due to their excellent performance, ease of application, and low cost [1]. However, the use of chromate conversion coatings was heavily restricted because of their high toxicity and carcinogenicity [2,3]. In response, various environmentally compatible coating systems have been developed to offer more options for the protection of aluminum alloys. 

Among these novel coating systems, layered double hydroxides (LDHs) appear to be a favorable alternative to the traditional chromate conversion coatings due to their characteristic structure and capability of ion exchange intercalation. With this unique capacity, LDHs have often been employed as containers for corrosion inhibitors such as rare earth ions to provide active corrosion protection for the metal substrates [4,5,6,7,8,9,10]. Zheludkevich et al. [4] prepared LDH-based nanocontainers with divanadate anions intercalated in the interlayer regions and found that the vanadate ions could be released in a controllable way, thus contributing to an obvious self-healing effect on the corrosion process and improved anti-corrosion properties. In addition to vanadate ions, a variety of other anions including molybdate(MoO_4_^2−^) [5,6], nitrate(NO_3_^−^), and carbonate(CO_3_^2−^) [7], and organic molecules such as polyaniline [8], quinaldate [9], and laurate [10], have also been intercalated in the interlayer gallery to promote the anticorrosive properties of the coating systems.

To further promote the properties of the films, LDH conversion films have been proposed in other studies [11,12,13,14,15,16,17,18,19,20,21,22,23,24,25,26]. LDH films were directly formed on the surface of a substrate, which could significantly enhance the adherence between the films and substrate. Furthermore, the LDH conversion films could be more compact and uniform due to the elimination of incompatibility between the LDH particles and the corresponding films. Since the pioneering work by Buchheit et al. [11,12,13], conversion coatings based on LDHs have experienced remarkable development, and significant advancements have been made through the newly developed systems [14,15,16,17,18,19,20,21,22,23,24,25]. Tedim et al. [15] grew LDH thin films directly on the surface of AA2024-T3 and long-term protection of AA2024-T3 was achieved when the specimens were immersed in a NaCl solution, especially when the specimens were intercalated with vanadate. In addition, very low current densities were detected, indicating an even higher level of protection. Chen et al. [25] reported a novel in situ hydrothermal method to fabricate an oriented Ni-Al-LDH film on a substrate, which turned out to be an excellent superhydrophobic coating material after simple superhydrophobic treatment. This coating is suitable for various applications (including anti-corrosion), combining the inherent features of the LDH films such as ion-exchange abilities. 

Despite all of the achievements mentioned above, LDH conversion films are still too thin to be applied in practice. Specifically, the LDH conversion films developed to date cannot provide sufficiently strong corrosion protection for practical applications. Further research is needed for such applications. On the other hand, sol-gel films have long been employed to protect aluminum alloys from corrosion due to their excellent barrier properties against corrosive media and their facile preparation procedures [26,27,28,29,30]. In this paper, we focused on the combination of LDH coatings and sol-gel films to fabricate a “smart” hybrid coating that could offer enhanced and active corrosion protection for substrates. 

## 2. Experiments

### 2.1. Pretreatment of Substrate 

The substrate used in this paper was AA2024-T3, and its chemical composition are displayed in Table 1.

There were two dimensions of specimens: 25 mm × 50 mm × 4 mm and 15 mm × 15 mm × 4 mm. The former was customized for scanning electron microscopy (SEM) observations, and the latter was used for other tests, including electrochemical tests and salt-spray test (SST). In addition, all the specimens were abraded with emery paper in the following sequence to remove the defects (such as scratches and dents) and grease produced during processing: #150→#240→#400→#800→#1500→#2000→#3000. Afterwards, the specimens went through ultrasonic cleaning with anhydrous ethanol and deionized water, respectively, to further cleanse the sample surface. Finally, the samples were dried in an oven and preserved in a drying vessel. 

### 2.2. Preparation of LDH Films 

In this paper, urea hydrolysis was used to fabricate the CO_3_·Mg-Al -LDH films. In detail, 0.903 g of magnesium sulfate (MgSO_4_), 1.283 g aluminum sulfate (Al_2_(SO4)_3_) and 3.574 g of urea (H_2_NCONH_2_) were dissolved in deionized water to form 100 mL of a mixed solution in which the concentration of Mg^2+^ and Al^3+^ was 0.075 mol/L, while that of urea was 0.595 mol/L. Afterwards, the proper amount of the solution was transferred into the hydrothermal reactor. The pretreated substrates were then immersed in the solutions mentioned above. To obtain the LDH films, the hydrothermal reactors were maintained at 90 °C for 1 h. The final step involved deionized washing and drying. In addition, the reaction temperature and time were varied (90 °C, 100 °C, 110 °C, and 120 °C and 1 h, 3 h, 5 h, 7 h and 9 h, respectively) to optimize the practical parameters. Moreover, the effect of the Mg^2+^ concentration was also investigated to obtain a more compact and uniform LDH film, evaluating Mg^2+^ concentrations of 0.038 mol/L, 0.075 mol/L, 0.113 mol/L, and 0.15 mol/L.

#### 2.2.1. Preparation of Decavanadate Anion-Intercalated LDH (LDH-V)

A decavanadate anion-intercalated LDH (LDH-V) film was prepared by anion exchange from precursor carbonate–containing LDHs. In detail, 12.2 g of NaVO_3_·2H_2_O was dissolved into 200 g of deionized water, and the pH of the obtained solution was adjusted to 4.5–5.5 with the appropriate addition of nitric acid (HNO_3_). The prepared specimens (covered by LDH films) were subsequently immersed in the solution for 24 h. After the reaction, the samples were washed with deionized water several times to clean the remaining solution and then air dried at ambient temperature.

#### 2.2.2. Preparation of the Sol

The sol-gel films used in this paper were prepared from precursors of tetraethyl orthosilicate (TEOS) and (γ-glycidyloxypropyl) trimethoxysilane (GPTMS). In detail, the molar ratio between TEOS and GPTMS was 1:2. Nitric acid (HNO_3_) and anhydrous ethanol (C_2_H_5_OH) were employed as the acid catalyst and solvent, respectively. In addition, the molar ratio between anhydrous ethanol and nitric acid was 0.75:1. Following the ratios mentioned above, anhydrous ethanol and nitric acid were first mixed together, followed by the addition of TEOS and GPTMS with stirring. After 3 h of stirring, the obtained sol was stored at 40 °C for 4 h. 

#### 2.2.3. Preparation of the Hybrid Coating (LDH-VS)

To prepare the hybrid coating (LDH-VS) composed of an LDH film and a sol-gel film, the obtained sol was used as prepared. Specifically, the hybrid coating was fabricated through the dip coating method using the specimen covered by the LDH film mentioned above. The detailed process was as follows: The specimen covered by the LDH film was first dropped into the sol at a speed of 300 µm/s and immersed in the sol for 1 min. Subsequently, the specimen was pulled out of the sol at a speed of 800 µm/s and exposed to air for 1 min. The whole process was repeated three times under the same conditions. Afterwards, the specimen was air dried at room temperature and then stored at 120 °C for 24 h, followed by air cooling at ambient temperature (25 °C). Finally, the obtained specimens were preserved in a drying vessel for future use. Substrates covered by the sol-gel coating (LDH-S) were also produced for comparison.

### 2.3. Surface Characterization and Other Tests

#### 2.3.1. Scanning Electron Microscopy (SEM)

The morphology of the coatings was investigated by SEM using an FEI Quanta 250 environmental scanning electron microscope (Field Electron and Ion Co. (FEI), Hillsboro, OR, USA) and an FEI Quanta 450 instrument (Field Electron and Ion Co. (FEI), Hillsboro, OR, USA). The latter can provide high-resolution images for elaborate analysis. In addition, both instruments were coupled with energy dispersive spectroscopy (EDS) and had an accelerating voltage of 20 kV. The vacuum chamber pressure was 2.8 × 10^−3^ Pa in the former and 4.5 × 10^−3^ Pa in the latter.

#### 2.3.2. X-ray Diffractometer (XRD) Analysis

The components of the coatings were analyzed on an XRD diffractometer (X’Pert Pro MPD, PANalytical, Almelo, The Netherlands) with CuKα radiation. The scanning rate and range were set at 10°/min and 5°–70°, respectively.

#### 2.3.3. Electrochemical Measurements

The electrochemical measurements were carried out with an electrochemical workstation (AutolabPGSTAT302N, Metrohm Autolab B.V., Utrecht, The Netherlands). A conventional three-electrode system was employed to conduct the tests, with a platinum plate, saturated calomel electrode (SCE) and the specimen acting as the counter, reference and working electrodes, respectively. In addition, the electrolyte used in these tests was a 3.5 wt % NaCl solution. Before the start of each test, the specimen was immersed in the electrolyte for 30 min for stabilization of the electrochemical system. Afterwards, electrochemical impedance spectroscopy (EIS) measurements were carried out over a frequency range 0.01–100 KHz. When the EIS test ended, potentiodynamic polarization tests were conducted at various voltage ranges coupled with different scanning rates for different specimens. For data processing, ZsimpWin (version 3.10, AMETEK Scientific Instruments, Berwyn, PA, USA) software and EC-Lab software (version 9.32, Bio-Logic Science Instruments, Seyssinet-Pariset, France) were used for the EIS data and potentiodynamic polarization data, respectively.

To study the corrosion of defective specimens in a corrosive medium (the electrolyte mentioned above), scanning electrochemical microscopy (SECM) measurements were conducted on a scanning electrochemical workstation (Princeton Applied Research, Model 370, Berwyn, PA, USA). The electrode system used in these tests was the same as that mentioned above, except that a microscopic platinum electrode with a diameter of 15 μm was also employed as part of the working electrode. In addition, the measurements were carried out at a speed of 15 μm/min, and the corresponding scanning area was 600 μm × 600 μm.

#### 2.3.4. Neutral SST

According to the ASTM B117 standard, SSTs were carried out using a salt spray chamber. The details of the operative conditions are as follows: Temperature-35 ± 2 °C; salt solution—5 wt % NaCl solution; humidity—95%.

#### 2.3.5. Ultraviolet-Visible Spectroscopy (UV-Vis) Measurements

UV-vis spectroscopy (by Perkin-Elmer 25 UV-vis spectrometer, Waltham, MA, USA) was used to investigate the release behavior of decavanadate ion.

## 3. Results and Discussion

### 3.1. Preparation of LDH Films

#### 3.1.1. XRD Patterns of LDH Films

**Figure 1:**
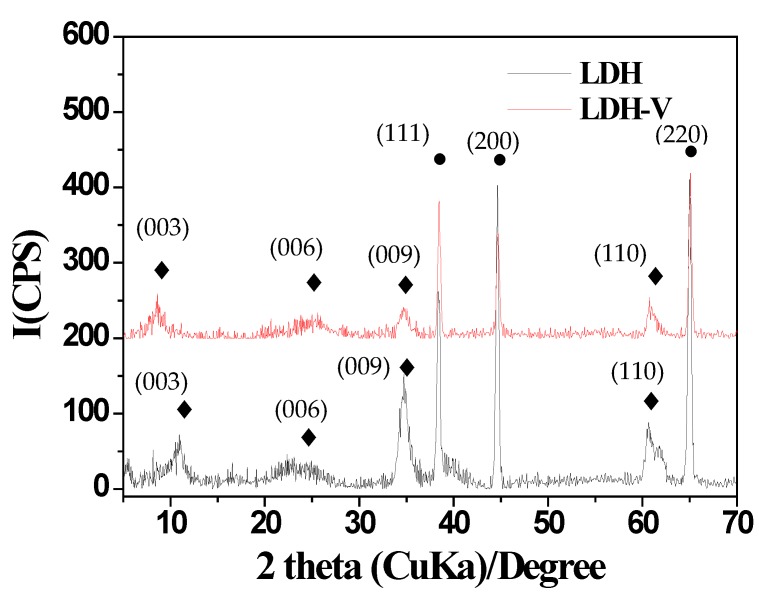
XRD Patterns of CO_3_·Mg-Al-LDH and LDH-V thin films: ♦ LDHs and LDHs-V; ● Al.

Figure 1 shows the XRD patterns of LDH and LDH-V films. In detail, reflections (003), (006), (009), and (110) indicate the presence of a CO_3_·Mg-Al-LDH film [31]. In addition, the shift of reflection (003) can be ascribed to the intercalation of decavanadate in LDH film-LDH-V [4]. Finally, the generation of reflections (111), (201), and (220) can be attributed to the substrate used [32].

#### 3.1.2. Effect of Practical Parameters on the Formation of LDH Films

**Figure 2:**
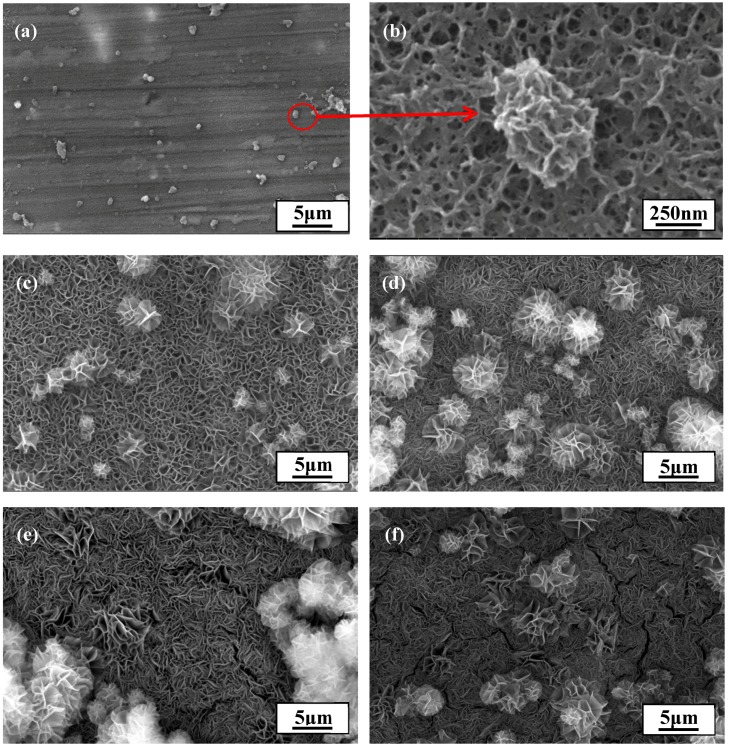
SEM images of LDH films formed at different reaction times: (**a**,**b**) 1 h; (**c**) 3 h; (**d**) 5 h; (**e**) 7 h; and (**f**) 9 h.

Practical parameters including the reaction time, Mg^2+^ concentration, and temperature have a remarkable influence on the formation of LDH films. Therefore, it is essential to optimize these parameters when fabricating films with excellent properties. First, the influence of reaction time was investigated while holding other parameters constant (Mg^2+^ concentration: 0.075 mol/L; temperature: 90 °C). The results are shown in Figure 2. The coating had hardly covered the surface of the substrate after 1 h of reaction (see Figure 2a), and a cellular structure was observed upon finer-scale observation (see Figure 2b). A reasonable explanation for this may be that the coating was in an early stage of nucleation when the reaction time reached 1 h. As the reaction time increased, the coating continued to grow and gradually covered the whole surface, producing a flower-like structure, at 3 h of reaction (see Figure 2c). Despite the covering, it was evident that the film was not compact enough, with lots of cracks remaining that could facilitate the permeation of corrosive ions such as Cl^−^. In other words, a longer reaction time was needed. When the reaction time reached 5 h, the coating obtained was evidently more compact, and defects such as cracks could hardly be found in the surface of the coating (see Figure 2d). Therefore, 5 h appeared to be the most appropriate reaction time for the formation of this film since serious defects such as large and deep cracks began to emerge when the reaction time was continuously increased to 7 h and 9 h (see Figure 2e,f).

**Figure 3:**
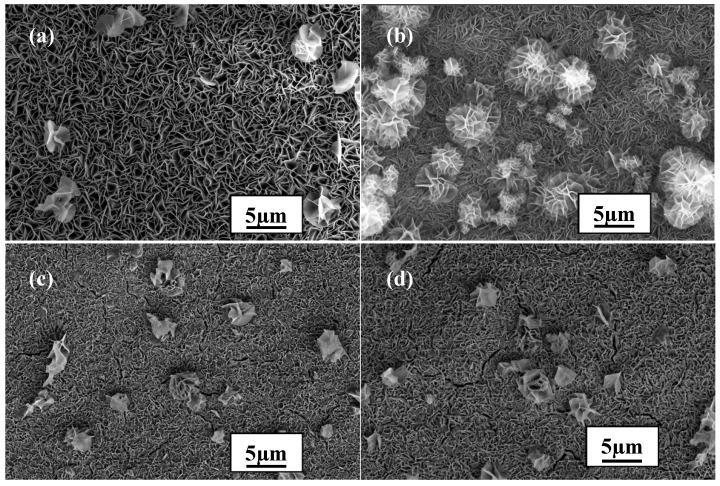
Surface morphologies of LDH films prepared with different concentrations of Mg2+: (**a**) 0.038 mol/L; (**b**) 0.075 mol/L; (**c**) 0.113 mol/L; and (**d**) 0.15 mol/L.

The concentration of Mg^2+^ also plays an essential role in the formation of an excellent coating. The influence of this factor was investigated by varying the concentration to 0.038 mol/L, 0.075 mol/L, 0.113 mol/L, or 0.15 mol/L, using the optimized reaction time of 5 h and maintaining the temperature at 90 °C. The corresponding results are displayed in Figure 3. When the concentration was low (0.038 mol/L), the amount of particles with flower-like structures was relatively low compared to that observed in Figure 2d (see Figure 3a). In addition, the particles were also obviously smaller, leaving numerous cracks between them, which indicates that the obtained coating was not very compact. However, when the concentration was increased to much higher values (0.113 mol/L and 0.15 mol/L), serious flaws (mostly cracks) appeared on the surface of the coating (see Figure 3c,d). Therefore, the optimal concentration was 0.075 mol/L (see Figure 3b). 

**Figure 4:**
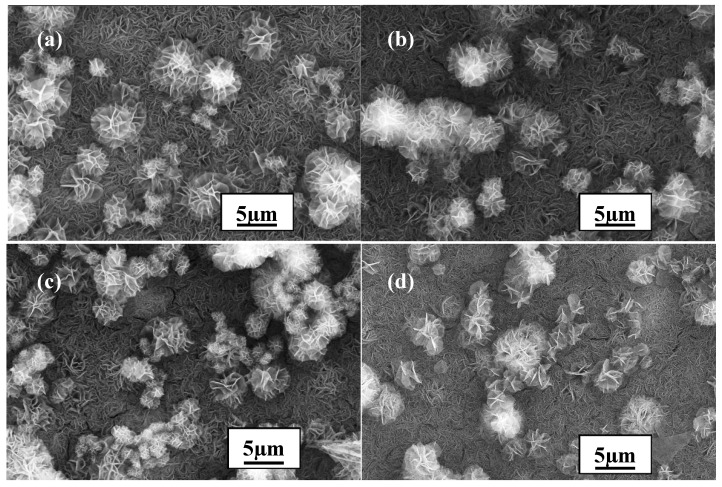
SEM diagrams of LDH films fabricated at different temperatures: (**a**) 90 °C; (**b**) 100 °C; (**c**) 110 °C; and (**d**) 120 °C.

The last parameter to be resolved was the temperature. To determine the most suitable temperature, it was varied between 90 °C, 100 °C, 110 °C and 120 °C, and the corresponding results are presented in Figure 4. The temperature clearly had a remarkable effect on the formation of the coating. Although the size of the crystalline particles did not change much, indicating that temperature had little effect on the size of the crystal particles, the specific structure of the particles transformed from curling to flattening (see Figure 4a–d). Furthermore, the crystalline degree of the particles obviously increased, indicating that the temperature had a significant influence on the crystallization. Despite the larger crystalline degree of the particles when the temperature was above 90 °C, the most suitable temperature for coating formation was still 90 °C since no evident defects such as cracks were found on the surface of the coating formed at this temperature compared to that formed at the other temperatures. 

In conclusion, the optimal reaction time, Mg^2+^ concentration, and temperature were determined to be 5 h, 0.075 mol/L, and 90 °C. All the subsequent coatings formed were based on these parameters.

### 3.2. Fabrication and Release Behavior of LDH-V Films

The LDH-V films were prepared by utilizing the inherent anion-exchange capability of the LDH films. UV-Vis spectroscopy was employed to confirm the successful intercalation of decavanadate.

**Figure 5:**
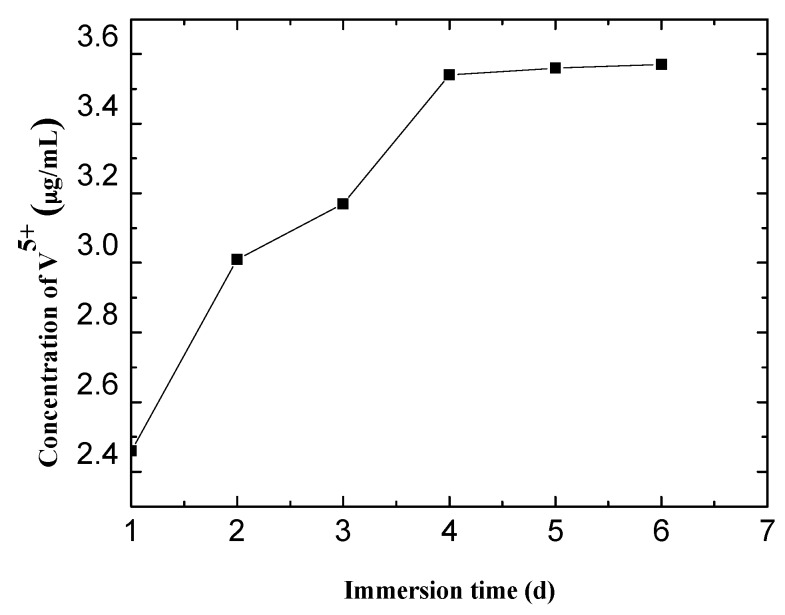
Release behavior of V^5+^ at different immersion times.

To investigate the release behavior of decavanadate in a corrosive medium, the LDH-V films were immersed in a 3.5 wt % NaCl solution for different periods of time. The results are displayed in Figure 5.

The release behavior of vanadium was then confirmed, meaning that decavanadate had been successfully intercalated in the LDH films. The release was relatively fast in the first 4 days with the rapid increase in the V^5+^ concentration in the solution. However, the release slowed down as vanadium reached saturation after 4 days, which means that the release and consumption of vanadium had reached a balance. 

**Figure 6:**
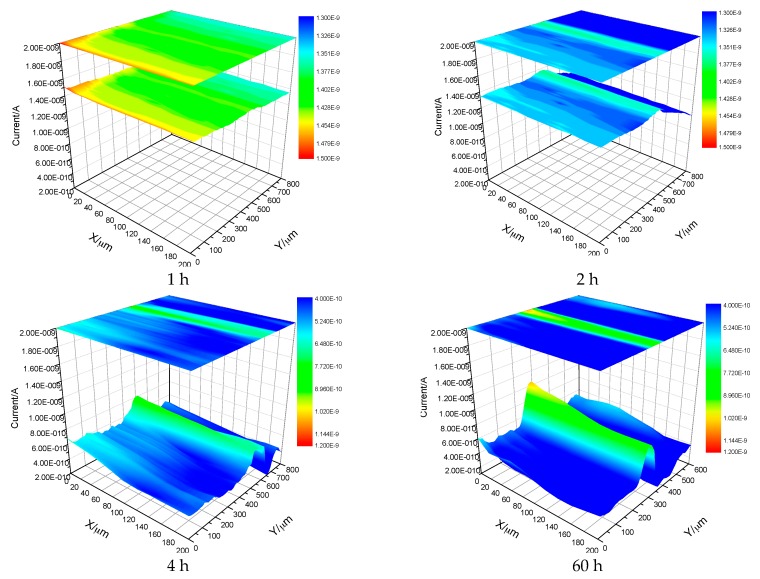
SECM images of scratched LDH-V films immersed in 3.5 wt % NaCl solution for different times.

To confirm the inhibiting effect of V^5+^ in corrosion behavior of LDH films, a SECM test was conducted on LDH-V film immersed in 3.5 wt % NaCl solution and the results were displayed in Figure 6. From Figure 6, we can see that the corrosive area was strictly restricted to the scratch due to the inhibition effect of V^5+^.

### 3.3. Preparation and Anti-Corrosive Behavior of the Hybrid LDH-VS Films

#### 3.3.1. Surface Characterization of the Hybrid Film

**Figure 7:**
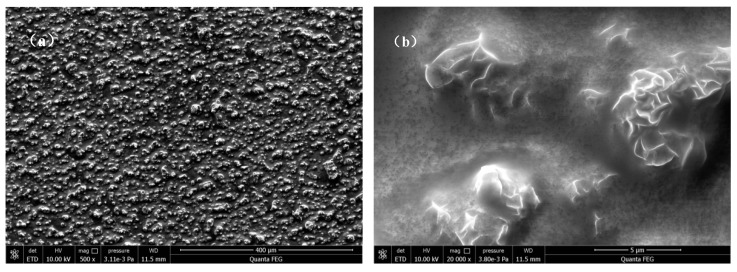
SEM images of an LDH-VS film: (**a**) low and (**b**) high magnification.

**Figure 8:**
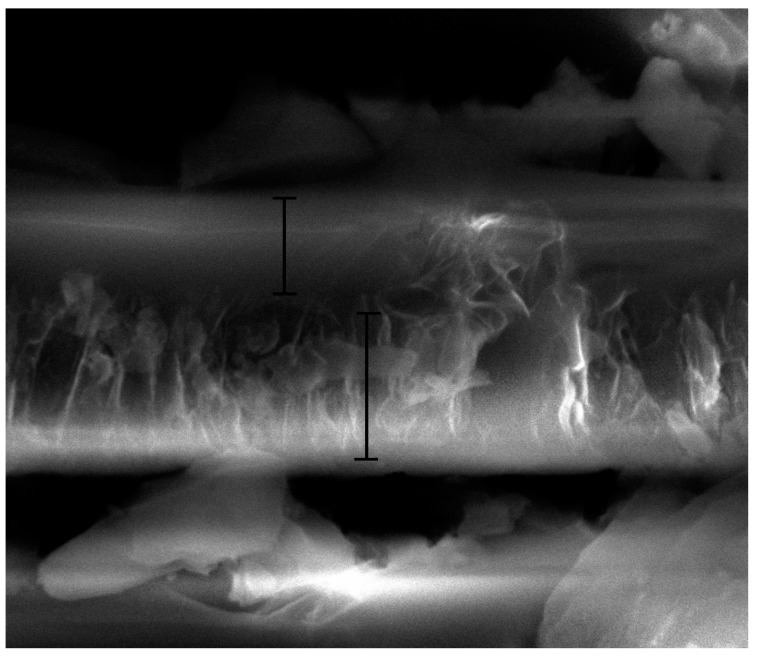
Cross-sectional image of an LDH-VS film.

**Figure 9:**
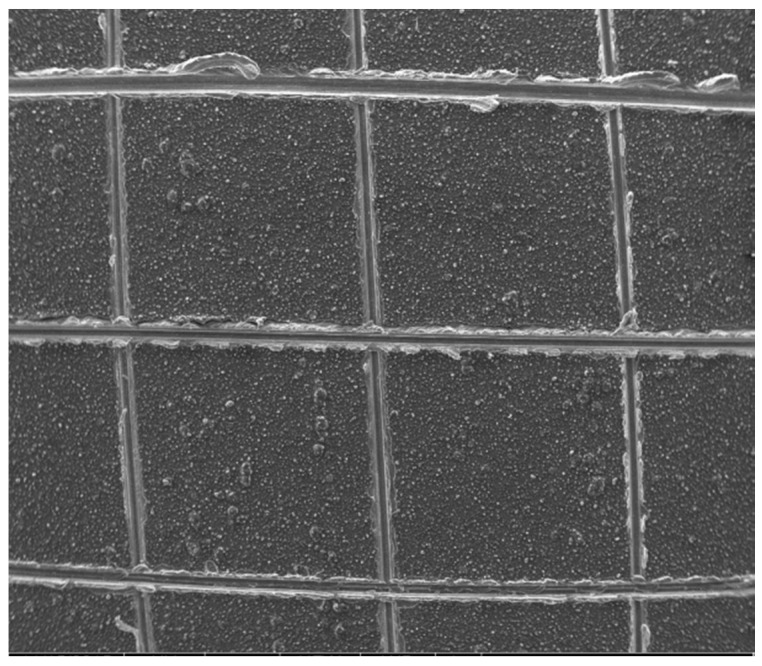
SEM image of the hybrid film during the scratch test.

Figure 7 shows the surface morphology of an LDH-VS film. As clearly shown in Figure 7a and Figure 9b, the surface of the LDH-V film was completely covered by the sol-gel film. Furthermore, the cracks in the LDH-V film were also permeated and covered by the sol-gel film, leading to a complete and defect-free coating (see Figure 7b). This finding was also verified by the cross-sectional image of the hybrid film (see Figure 8) which clearly showed that the sol-gel film had completely covered the LDH-V film, including defects such as cracks in the film. Moreover, the thickness of each film could also be obtained from Figure 8: the thickness of the LDH-V film was approximately 3.5 μm, and the thickness of the sol-gel film was approximately 2.3 μm. 

#### 3.3.2. Adhesion Test of the Hybrid Coating

According to the GBT9286-1998 standard, the cross-cut method was used to measure the adhesion between the LDH-V film and substrate. In addition, the adhesive force between the LDH-V film and sol-gel film could also be tested in this experiment. The corresponding result is presented in Figure 9. As revealed in the figure, no obvious exfoliation was found on the edges of every grid cut out on the surface of the hybrid film. The adhesion was rated as zero grade, indicating excellent adhesion between the films. This excellent adhesion would greatly benefit the anti-corrosive property of the coating since it would make penetration of the film by corrosive media very difficult. 

#### 3.3.3. Corrosion-Resistant Properties of the Hybrid Coatings

**Figure 10:**
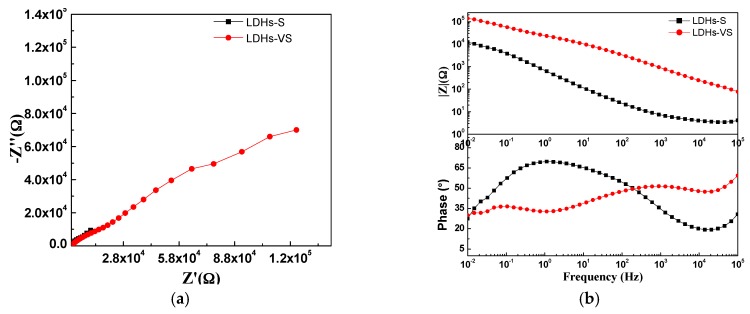
EIS test of the sol-gel coating and hybrid film in a 3.5 wt % NaCl solution: (**a**) Nyquist plot; and (**b**) Bode plot.

**Figure 11:**
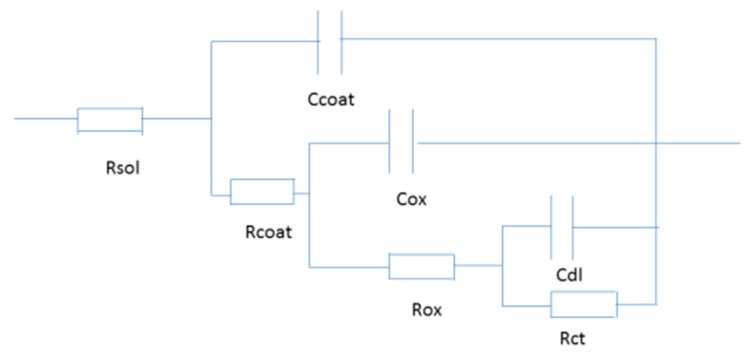
Equivalent circuit for the EIS fitting in Figure 10, Figure 12 and Figure 13.

To investigate the anti-corrosive performance of the hybrid coatings (LDH-VS), EIS was used to evaluate the impedance of these films in a corrosive medium (3.5 wt % NaCl solution). For comparison, the LDH-S films were also prepared. The corresponding results are presented in Figure 10. These results were also furthered analyzed by ZsimpWin (Version 3.30), and the fitting results are shown in Table 2. Importantly, the fitting process mentioned above was conducted with reference to the equivalent circuit represented in Figure 11, in which Rsol, Rcoat, Rox, and Rct represent the resistance of the solution, coating, oxide layer, and charge transfer, respectively, and Ccoat, Cox, and Cdl represent the capacitance of the coating, oxide layer, and double layer, respectively. It must be pointed out that no obvious dispersion effect was observed according to the EIS results obtained. That is why capacitance rather than Constant Phase Element (CPE) was used here. Therefore, the corresponding *n* value should be 1.

The hybrid film (LDH-VS) had a much larger impedance than that of LDH-S (see Figure 10), indicating that the composite film (LDH-VS) was the best at protecting the substrate from corrosion. This was also verified by the fitting results displayed in Table 2, in which the coating resistance (Rcoat) increased from 2.4 × 10^2^ Ω for LDH-S to 1.6 × 10^3^ Ω for LDH-VS, while the capacitance of the coating (Ccoat) decreased abruptly from 1.0 × 10^−4^ F for LDH-S to 8.0 × 10^−8^ F for LDH-VS (see Table 3). These results were probably due to the dual effect of decavanadate on the prevention of corrosion. On the one hand, corrosive media such as chlorine ions (Cl^−^) were trapped in the LDH structure through ion exchange. On the other hand, the decavanadate released through ion exchange played a key role in the inhibition of corrosion. In addition, the hybrid film presented three typical time constants that corresponded to the electrochemical reactions of the hybrid coating, electrical double layer, and decavanadate, respectively (see Figure 10b). In contrast, the LDH-S film only displayed two typical time constants corresponding to the electrochemical reactions of the sol-gel film and electrical double layer, respectively. In summary, these data further confirmed the effect of decavanadate on the corrosion protection of the film.

**Figure 12:**
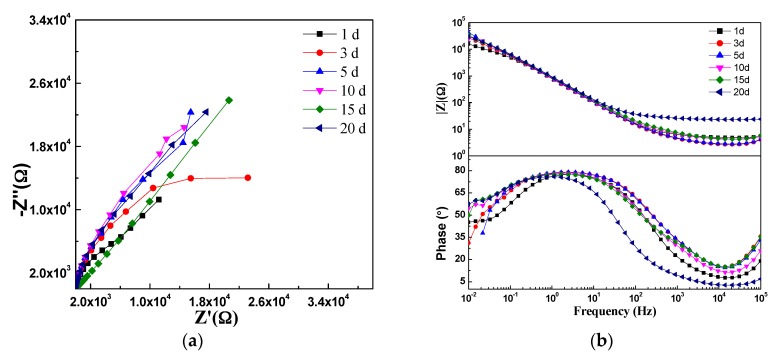
EIS plot of the LDH-S film after immersing in a 3.5 wt % NaCl solution for different time periods: (**a**) Nyquist plot; and (**b**) Bode plot.

**Figure 13:**
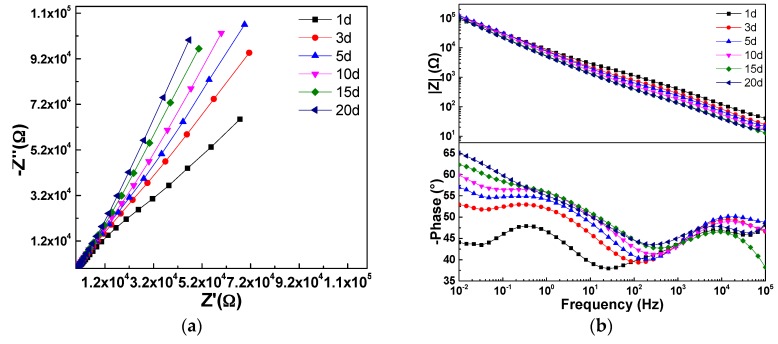
EIS plot of the LDH-VS film after immersing in a 3.5 wt % NaCl solution for different time periods: (**a**) Nyquist plot; and (**b**) Bode plot.

To further investigate the anti-corrosive properties of LDH-VS and LDH-S, immersion tests were carried out in a 3.5 wt % NaCl solution, and the results are displayed in Figure 12 and Figure 13, respectively. For LDH-S, the impedance did not change much in the first 15 days (see Figure 12). However, the impedance slightly increased when the immersion time reached 20 days, which was probably due to the corrosion product produced during the corrosion process. In addition, the number of time constants had also declined from 2 to 1 when the immersion time reached 20 days, indicating that a remarkable change had occurred during that time. The exact explanation for this phenomenon may be that the sol-gel coating had completely failed and that the electrochemical reactions related to it simply disappeared. In contrast, these changes did not occur in the hybrid film. As shown in Figure 13, the impedance of the hybrid film did not change much during the whole immersion test, meaning that the function of the coating remained normal during the test. This was further verified by the Bode plot (see Figure 13b) of the coating, which showed that the number of time constants also remained unchanged. Therefore, it was proposed that LDH-VS still functioned well even after 20 days of immersion in electrolyte, which was ascribed to the synergistic effect of the LDH-VS film.

The proposition mentioned above was further confirmed by the fitting results shown in Table 3 and Table 4. For instance, Table 3 shows that the coating resistance of LDH-VS was highest (7.2 × 10^1^ Ω) when the immersion time was 1 day and subsequently fluctuated from 1.0 × 10^1^ Ω to 2.7 × 10^1^ Ω. In contrast, the coating resistance of LDH-VS varied from 2.5 × 10^2^ Ω to 6.3 × 10^2^ Ω (see Table 4), which is a much larger variation than that of LDH-S. Furthermore, the coating resistance of LDH-VS remained as high as 2.8 × 10^2^ Ω at the end of the immersion test, compared to 2.7 × 10^1^ Ω for LDH-S. Furthermore, the coating capacitance of LDH-VS was generally two orders lower than that of LDH-S. Although the coating capacitance of LDH-VS continued to increase throughout the entire test, the final value was less than 5.8 × 10^−7^ F, which is approximately two magnitudes lower than that of LDH-S (4.2 × 10^−5^ F). Finally, the above proposition could also be validated by the charging resistance (Rct). In general, the charging resistance of LDH-VS was one order of magnitude higher than that of LDH-S. 

**Figure 14:**
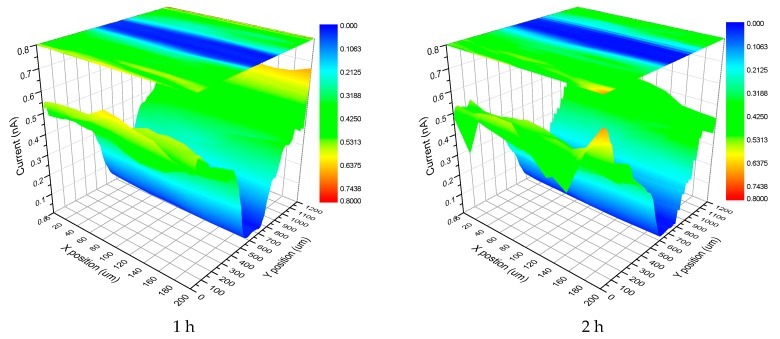

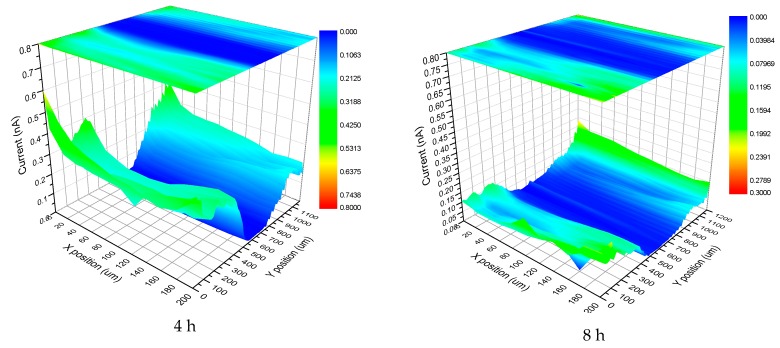
SECM images of the LDH-S film immersed in a 3.5 wt % NaCl solution for different time periods.

**Figure 15:**
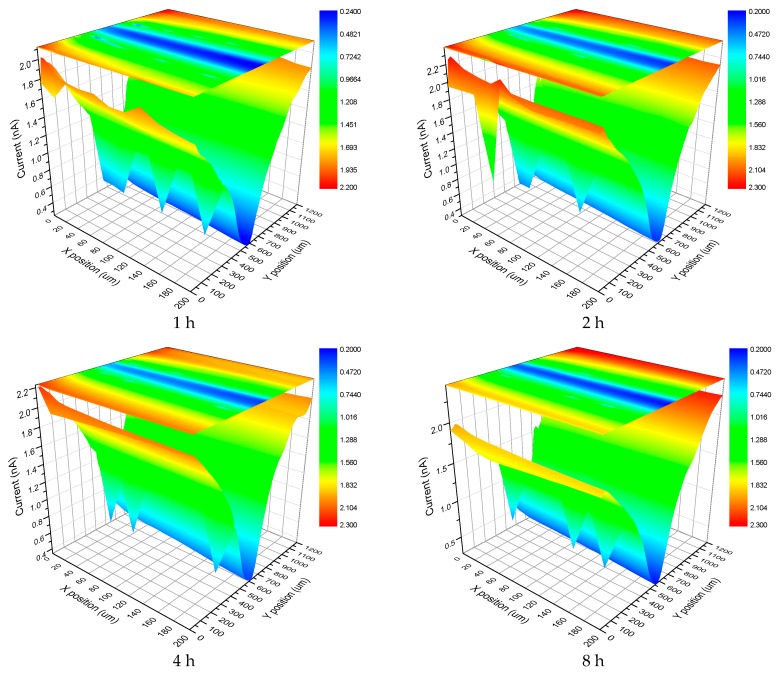
SECM images of the LDH-VS film immersed in a 3.5 wt % NaCl solution for different time periods.

Scanning Electrochemical Microscopy (SECM) was also used to investigate the corrosion of the films. The results are presented below. Figure 14 shows the SECM results of the LDH-S film immersed in 3.5 wt % NaCl for different time periods. It must be pointed out that the current in Figure 14 and Figure 15 is actually the tip current, not the corrosion current on the surface of the sample. The results perfectly display the evolution of the corrosion process during immersion by measuring the local current of a man-made scratch on the surface of the sol-gel film. In the first hour of immersion, the corrosion was limited to the scratch and its nearby section. As the immersion time increased to 2 h, the corrosive section only expanded laterally to the nearby area. Subsequently, this trend continued until the end of test. In contrast, the process in the LDH-VS film was completely different. Instead of continued lateral expansion along the surface of the film, the corrosive area remained constant during the entire test (see Figure 15), which was probably attributed to the unique characteristics of the LDH-VS film (synergic effect of the LDH-V film).

**Figure 16:**
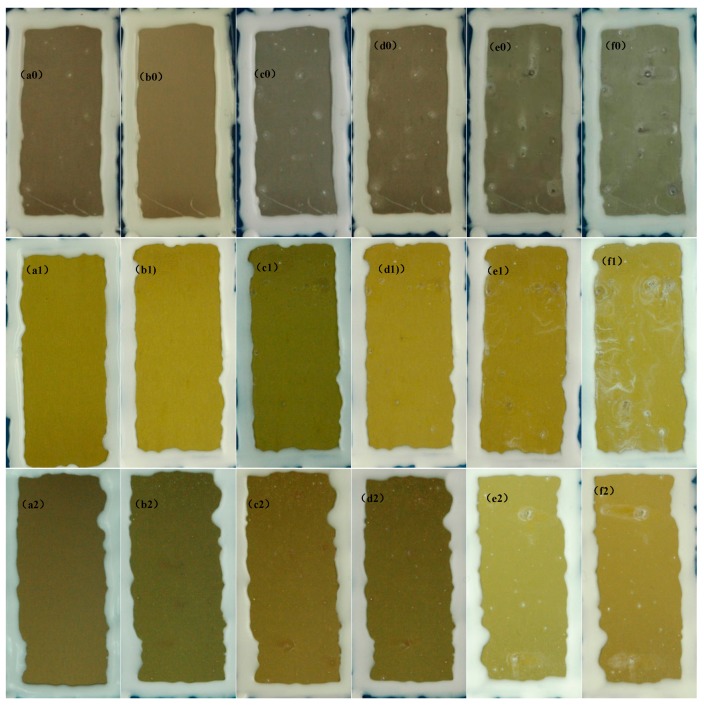
SST results for the LDH-S (**a0**–**f0**), LDH-V (**a1**–**f1**) and LDH-VS (**a2**–**f2**) films: (**a**) 0 day; (**b**) 1 day; (**c**) 2 day; (**d**) 3 day; (**e**) 7 day; and (**f**) 10 day.

As shown in Figure 16, the corrosion-resistant property of the LDH-VS film was significantly superior to that of the LDH-S film. Specifically, obvious white pits (corrosion product) were observed only after 1 day of the test (see Figure 16b0). Moreover, the performance of LDH-S deteriorated rapidly as the test time increased (see Figure 16c0–f0). At the end of the test (10 days), the film appeared to have completely failed. In contrast, the LDH-VS exhibited excellent anti-corrosive performance during the test (see Figure 16a2–f2). Although a few white pits were observed after 1 day of testing, the corrosion process was rigorously controlled in the later stage of the test, which could have been attributed to the intercalation of decavanadate in the LDH structure. In addition, this result perfectly corresponds to the electrochemical measurements (including the macroscopic and microscopic measurements).

The effect of the addition of the sol-gel film on the corrosion protection ability was also confirmed by comparing the LDH-V film with the LDH-VS film (see Figure 16a1–f1 and Figure 16a2–f2). In the short term (during the first 3 day of testing), the LDH-V film showed superior anti-corrosive performance to that of the LDH-VS film, probably due to the corrosion of the sol-gel film at the start of the test. However, the LDH-VS film exhibited better performance in the long term (see Figure 16f1,f2), which could be ascribed to the defect-free structure resulting from the filling of the sol gel (see Figure 7). In other words, these data were in good agreement with the SEM observations.

## 4. Conclusions

The optimal practical parameters for the preparation of LDH films were as follows: reaction time of 5 h, Mg^2+^ concentration of 0.075 mol/L, and temperature of 90 °C. In addition, the LDH-VS film formed on the substrate was approximately 5.843 μm thick. By depositing a sol gel on the surface of the LDH-V film, defects such as cracks existing inside the LDH-V film were eliminated, significantly contributing to the improvement in the anti-corrosion performance of the LDH-VS film. Furthermore, the adhesion of the LDH-VS film was also enhanced by the surface characteristics of the hybrid sol-gel film, which could facilitate the application of other organic coatings. On the other hand, the intercalation of decavanadate in the LDH film also greatly improved the corrosion-resistant property of the LDH-VS film, especially the long-term corrosion protection, which was probably due to the synergistic effect of the film. 

## Figures and Tables

**Table 1:** Chemical composition of AA2024-T3.

Element	Cu	Mg	Mn	Fe	Si	Zn	Cr	Al
Content (wt %)	4.35	1.36	0.67	0.18	0.10	0.07	0.02	Balance

**Table 2:** EIS fitting results for Figure 10.

Type of LDH Films	Rsol/Ω	Ccoat/F	Rcoat/Ω	Cox/F	Rox/Ω	Cdl/C	Rct/Ω	Chi-Squared
LDH-S	2.4 × 10^1^	1.0 × 10^−4^	2.4 × 10^2^	9.7 × 10^−5^	6.2 × 10^3^	9.3 × 10^−4^	1.5 × 10^4^	7.7 × 10^−3^
LDH-VS	1.1 × 10^2^	8.0 × 10^−8^	1.6 × 10^3^	6.4 × 10^−7^	1.8 × 10^4^	2.2 × 10^−5^	1.0 × 10^5^	1.2 × 10^−1^

**Table 3:** EIS fitting results for Figure 12.

Immersion time/day	Rsol/Ω	Ccoat/F	Rcoat/Ω	Cox/F	Rox/Ω	Cdl/C	Rct/Ω	Chi-Squared
1	5.5 × 10^0^	9.2 × 10^−5^	7.2 × 10^1^	9.6 × 10^−5^	6.6 × 10^3^	6.9 × 10^−4^	1.8 × 10^4^	2.3 × 10^−2^
3	3.2 × 10^0^	6.2 × 10^−5^	1.5 × 10^1^	9.3 × 10^−5^	2.2 × 10^3^	1.4 × 10^−4^	2.2 × 10^4^	5.0 × 10^−2^
5	5.4 × 10^0^	5.8 × 10^−5^	2.0 × 10^1^	1.0 × 10^−4^	5.7 × 10^3^	2.2 × 10^−4^	3.6 × 10^4^	2.3 × 10^−2^
10	4.6 × 10^0^	4.5 × 10^−5^	1.7 × 10^1^	9.8 × 10^−5^	2.8 × 10^3^	1.4 × 10^−4^	3.3 × 10^4^	3.1 × 10^−2^
15	2.0 × 10^-5^	3.6 × 10^−7^	1.0 × 10^1^	4.6 × 10^−5^	7.4 × 10^2^	1.1 × 10^−4^	2.2 × 10^4^	3.6 × 10^−1^
20	8.4 × 10^0^	4.2 × 10^−5^	2.7 × 10^1^	1.0 × 10^−4^	5.3 × 10^3^	2.0 × 10^−4^	3.5 × 10^4^	3.4 × 10^−2^

**Table 4:** EIS fitting results for Figure 13.

Immersion time/day	Rsol/Ω	Ccoat/F	Rcoat/Ω	Cox/F	Rox/Ω	Cdl/C	Rct/Ω	Chi-Squared
1	4.9 × 10^1^	1.6 × 10^−7^	6.3 × 10^2^	2.4 × 10^−6^	4.8 × 10^3^	3.3 × 10^−5^	8.3 × 10^4^	1.2 × 10^−1^
3	3.1 × 10^1^	2.3 × 10^−7^	5.7 × 10^2^	5.5 × 10^−6^	7.0 × 10^3^	4.1 × 10^−5^	1.2 × 10^5^	1.2 × 10^−1^
5	2.5 × 10^1^	2.7 × 10^−7^	4.6 × 10^2^	6.0 × 10^−6^	6.9 × 10^3^	4.2 × 10^−5^	1.5 × 10^5^	1.2 × 10^−1^
10	2.0 × 10^1^	3.5 × 10^−7^	3.3 × 10^2^	6.9 × 10^−6^	5.7 × 10^3^	4.6 × 10^−5^	1.5 × 10^5^	1.2 × 10^−1^
15	1.7 × 10^1^	5.0 × 10^−7^	2.5 × 10^2^	8.2 × 10^−6^	4.7 × 10^3^	5.6 × 10^−5^	1.5 × 10^5^	1.1 × 10^−1^
20	1.9 × 10^1^	5.8 × 10^−7^	2.8 × 10^2^	8.9 × 10^−6^	4.8 × 10^3^	6.2 × 10^−5^	1.7 × 10^5^	1.2 × 10^−1^
